# Development of the expression and prognostic significance of m^5^C‐related LncRNAs in breast cancer

**DOI:** 10.1002/cam4.5500

**Published:** 2022-12-04

**Authors:** Gaoran Xu, Chengxin Li, Ziyang Di, Yalong Yang, Leilei Liang, Qianqian Yuan, Qian Yang, Xingxing Dong, Siguang Xu, Gaosong Wu

**Affiliations:** ^1^ Department of Thyroid and Breast Surgery Zhongnan Hospital of Wuhan University Wuhan China; ^2^ Department of Gastrointestinal Surgery and Department of Gastric and Colorectal Surgical Oncology Zhongnan Hospital of Wuhan University Wuhan China; ^3^ Department of Gynecologic Oncology, National Cancer Center/National Clinical Research Center for Cancer/Cancer Hospital Chinese Academy of Medical Sciences and Peking Union Medical College Beijing China; ^4^ Xinxiang Medical College Xinxiang Medical University Xinxiang China

**Keywords:** breast cancer, drug sensitivity, lncRNA, m5C, risk score signature, tumor immune microenvironment

## Abstract

**Background:**

5‐Methylcytosine (m5C) methylation is a major epigenetic RNA modification and is closely related to tumorigenesis in various cancers. This study aimed to explore the prognostic value of m5C‐related lncRNAs in breast cancer.

**Methods:**

Clinical characteristics and RNA‐seq expression data from TCGA (The Cancer Genome Atlas) were used in the study. First, we performed differentially expressed gene (DEG) analysis and constructed a PPI network for the 12 m5C regulators. Then, we identified the m5C‐related LncRNAs by the “cor. test.” An m5C‐related lncRNA prognostic risk signature was developed using univariate Cox regression and Lasso‐penalized Cox regression analyses. The model's performance was determined using Kaplan–Meier (KM) survival analysis and ROC curves. Finally, a nomogram was constructed for clinical application in evaluating patients with BRCA. We also researched the drug sensitivity of signature lncRNAs and immune cell infiltration. Finally, we validated the expression of the signature lncRNAs through qRT–PCR in a breast cancer cell line and a breast epithelial cell line.

**Results:**

Overall, we constructed an 11‐lncRNA risk score signature based on the lncRNAs associated with m5C regulators. According to the median risk score, we divided BRCA patients into high‐ and low‐risk groups. The prognostic risk signature displayed excellent accuracy and demonstrated sufficient independence from other clinical characteristics. The immune cell infiltration analysis showed that the prognostic risk signature was related to the infiltration of immune cell subtypes. Drug sensitivity proved that our prognostic risk signature potentially has therapeutic value.

**Conclusions:**

The m5C‐related lncRNA signature reliably predicted the prognosis of breast cancer patients and may provide new insight into the breast cancer tumor immune microenvironment.

## Graphical abstract

By RNA‐seq and TCGA, an 11‐lncRNA risk score signature was constructed based on lncRNAs associated with m5C regulators, which could reliably predict the prognosis of breast cancer patients. Immune cell infiltration assays and drug susceptibility assays demonstrate their value for studying the tumor immune microenvironment in breast cancer.

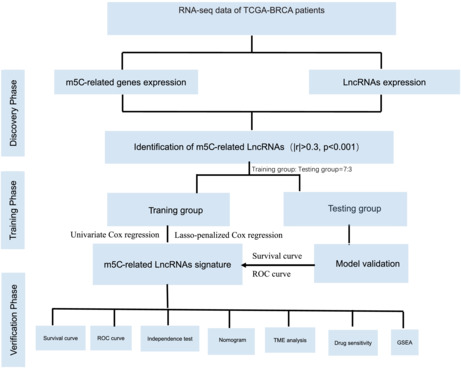

## 1 BACKGROUND

Breast cancer (BRCA) is one of the most common malignancies among women worldwide.^1^, ^2^ In 2018, breast cancer accounted for 24.2% of all newly diagnosed malignant tumors in women and 15% of all malignant tumor‐associated deaths.^3^ Although the pathogenesis of breast cancer is not completely understood, multiple pathways and molecular mechanisms have been proposed.^1^, ^3^, ^4^ Many genetic changes have been identified to be associated with the development and progression of breast cancer.^5^, ^6^ However, the role of epigenetic changes in breast cancer pathogenesis has not been well examined.

Epigenetic changes, such as methylation modification and histone modifications, can reversibly and heritably modify gene function without interfering with DNA sequences.^7^ In addition to DNA, RNA has also been found to be modified by epigenetic modification. Since the discovery of the first RNA modification almost 60 years ago in yeast, over 150 additional modifications have been identified in all types of RNA.^8^ It is known that epigenetic changes may induce various diseases, including cancer.^6^, ^9^

The internal modification of mRNA was first discovered in the form of N6‐methyladenosine (m6A) several years ago,^10^ and it remains the most abundant internal modification for mRNA and ncRNA. Apart from m6A modifications, accumulating evidence suggests that N1‐methyladenosine (m1A), pseudouridine (ψ), and 5‐methylcytosine (m5C) are also important formats of RNA posttranscriptional epigenetic regulation.^11^, ^12^ In particular, m5C is one of the most novel modifications that has been reported to be detected not only in ribosomal RNA and transfer RNA but also in messenger RNA.^13^, ^14^ Dysregulated m5C modification can result in impaired RNA metabolism and contribute to the pathogenesis of various diseases.^15^, ^16^, ^17^ LncRNAs account for 80% of ncRNAs and play important roles in various cellular functions by participating at multiple regulatory levels (transcriptional, posttranscriptional, translational, posttranslational, and epigenetic). In general, the associations between long noncoding RNA (lncRNA) m5C changes and human diseases, especially breast cancer, are largely unexamined.^18^, ^19^ This study aimed to describe the epigenetic changes in breast cancer tissue and explore the relationship with lncRNAs.

## 2 METHODS

### 2.1 Data acquisition and preparation

Transcriptome sequencing data of mRNAs and lncRNAs with corresponding patients' clinical information of 1222 samples, including 113 adjacent noncancerous samples and 1109 tumor samples, were acquired from the TCGA‐BRCA databank (https://portal.gdc.cancer.gov/). After excluding data without identified survival outcomes, a total of 1069 BRCA patients' clinical characteristic details were collected for this study and are presented in Table 1. These patients were randomly assigned to the training group and testing group (Table S1). For external validation, we obtained the GEO dataset GSE20685, which includes 327 breast cancer patients. We downloaded the gtf. annotation file (GRCh38.p12) from the GENCODE database (http://www.gencodegenes.org) and extracted the Ensemble IDs of lncRNAs in the TCGA database after screening 13,162 lncRNAs. After a literature review, 12 m5C regulators, including *NSUN2*, *NSUN3*, *NSUN4*, *NSUN5*, *NSUN6*, *NSUN7*, *DNMT1*, *DNMT3A*, *DNMT3B*, *TET2*, *TRDMT1*, and *ALYREF*, were selected for further analysis. Next, we conducted differentially expressed gene (DEG) analysis using the R software (version 4.0.3) “*limma*” package. The threshold of DEGs was set as |log_2_Fold Change| ≥ 1 and *p* value <0.05. The packages “*vioplot*” and “*pheatmap*” were applied to plot the vioplot and heatmap. The R software package “limma” was also used to screen the m5C‐related lncRNAs. The correlation between 12 m5C regulators and 13,162 lncRNAs was tested by the “cor.test.” The m5C‐related lncRNAs with |correlation coefficient| >0.3 and *p* value <0.001 were selected for further analysis.

**TABLE 1:** Clinical characteristic of TCGA‐BRCA patients

Clinical characteristic	TCGA‐BRCA (*n* = 1069)	Percentage
Survival status		
Alive	921	86.20
Dead	148	13.80
Age		
<65	747	69.90
≥65	322	30.10
Pathologic stage		
Stage I	181	16.90
Stage II	606	56.70
Stage III	240	22.40
Stage IV	20	1.90
Unknow	22	2.10
T		
T1	279	26.10
T2	617	57.70
T3	132	12.30
T4	38	3.60
TX	3	0.30
N		
N0	502	47.00
N1‐3	550	51.40
NX	17	1.60
M		
M0	890	76.70
M1	22	2.00
MX	157	21.30

### 2.2 Bioinformatic analysis

The STRING database (Version 11.0 http://www.string‐db.org) was used to construct a protein–protein interaction network with a minimum required interaction score of 0.7 (high confidence). Then, Pearson's correlation coefficient analysis was applied to calculate the associations between m5C regulators, which were visualized by the R package corrplot v.0.84. To identify the prognostic value of m5C‐related lncRNAs, univariate Cox regression analysis was performed. Then, the hazard ratio (HR) with 95% confidence interval of each m5C‐related lncRNA was calculated, and *p* value <0.01 was set as the threshold of significant difference. The R packages “caret” and “glmnet” were used to construct the prognostic signature. Based on the Lasso‐penalized Cox regression analysis, the prognosis‐relevant lncRNAs were used to construct the prognostic signature in the training group. The risk score was calculated for each training group patient as follows: risk score = $\sum_{i=1}^{n}\left(\text{Coefi}*\text{Expi}\right)$, where Coefi stands for the regression coefficient of lncRNAs and Expi stands for the relative expression values of lncRNAs. m5C‐related lncRNAs with *Coefi >*0 were defined as the risk signature, and those with *Coefi* <0 were defined as the protective signature. The median risk score was set as the cutoff point to determine the low‐ and high‐risk patients in the training group. Next, patients in the testing group were assigned to the high‐risk group and the low‐risk group depending on the comparison of their risk scores to the median risk score obtained in the training group. Kaplan–Meier and time‐dependent ROC analyses were performed to validate the efficacy of the signature, and the R packages “survival,” “survminer,” and “timeROC” were used for the analysis. Univariate Cox regression analysis and multivariate Cox regression analysis were performed to identify the independent risk factors, and a *p* value <0.05 was considered to indicate significant difference. A nomogram was then constructed to enable clinicians to conveniently use our prognostic model in evaluating 3‐ and 5‐year overall survival (OS) with risk factors identified from the multivariate Cox regression analysis by the R packages “rms” and “survival.” The prognostic value of the nomogram was further validated by calibration curve and c‐index analyses.

### 2.3 Evaluation of immune cell infiltration

CIBERSORT is a tool for deconvoluting the expression matrix of human immune cell subtypes based on the principle of linear support vector regression. We used the CIBERSORT algorithm to determine the abundances of tumor‐infiltrating immune cells (TIICs), including seven T‐cell subsets, two B‐cell subsets, plasma cells, NK cells (activated and resting), dendritic cells (activated and resting), mast cells (activated and resting), eosinophils, neutrophils, and monocyte macrophages (M0‐M2). The infiltration of CD4 naive T cells was not measured in this study due to the extremely low abundance or complete absence of that cell type. Wilcoxon's rank‐sum test was used to visualize the immune cell infiltration in the high‐ and low‐risk groups by violin plot. The correlation between immune cells and immune/stromal/ESTIMATE scores was evaluated using Spearman correlation analysis. Finally, we examined the association between m5C‐related risk factors and immune cell infiltration.

### 2.4 Chemotherapy response with the m5C‐related lncRNA signature

Patient chemotherapy treatment data were downloaded from the Genomics of Drug Sensitivity in Cancer (*GDSC*) dataset (http://www.cancerrxgene.org/downloads/). The R package “pRRophetic” was utilized to estimate the drug sensitivity for each BRCA patient by calculating half maximal inhibitory concentration (IC50) levels, and Wilcoxon's rank‐sum test was performed to compare the statistics.

### 2.5 Gene set enrichment analysis (GSEA)

GSEA was conducted based on the C2.CP.KEGG.v7.4 gene set using GSEA software (version 4.1.0). Statistical significance was set as NOM *p* < 0.05 and FDR <0.25.

### 2.6 External validation of the prognostic value of the lncRNA WEE2‐AS1

To validate our m5C lncRNA signature, we annotated the GSE20685 dataset with the annotation downloaded from the GEO website (https://www.ncbi.nlm.nih.gov/geo/query/acc.cgi?acc=GSE20685). Only the lncRNA WEE2‐AS1 was found in the GSE20685 dataset, so we decided to validate the prognostic value of WEE2‐AS1. The independence of the lncRNA WEE2‐AS1 was evaluated by univariate Cox regression analysis and multivariate Cox regression analysis. We also compared our m5C‐lncRNA signature with other breast cancer signatures based on the TCGA dataset. The C‐index was used to present the prediction accuracy.

### 2.7 Cell culture

The human normal breast epithelial cell line MCF‐10A and the breast cancer cell lines MDA‐MB‐231 and MCF‐7 were obtained from Procell. The MCF‐10A cell line was cultured in MCF‐10A specialized medium CM‐0525 (Procell). Other cell lines were cultured in DMEM (Irvine Scientific) supplemented with 10% fetal bovine serum (FBS, Gibco, USA).

### 2.8 RNA extraction and qPCR analysis

RNA was extracted from cells using a Hipure Total RNA Mini Kit (R4111‐03, Magen, China). A HiScript II QRT SuperMix (Vazyme, China) kit was used for reverse transcription. qRT–PCR was conducted using the SYBR GREEN MIX (Vazyme, China) kit by the CFX96 Real‐time PCR Detection System (Bio‐Rad). GAPDH was selected as the internal housekeeping gene, and the relative gene expression was calculated by the 2^−ΔΔCt^ method. Each qRT–PCR was repeated three times. The primary results are shown in Table S2.

### 2.9 Statistical analysis

The statistical analysis was conducted by R (version 4.0.3) and Perl (version 5.24) software. Random sequences were generated by SPSS 22.0. To improve the accuracy and reduce the number of lncRNAs in the prognostic signature, a *p* value <0.01 was considered to indicate a significant difference in the univariate Cox regression analysis. Survival curves were evaluated by Kaplan–Meier analysis and the log‐rank test, and a *p* value <0.05 was considered to indicate a significant difference. ROC curves were plotted to assess the prediction accuracy of the prognostic signature, with AUCs of 0.6–0.7, 0.7–0.9, and 0.9–1.0 representing acceptable, moderate, and high accuracy, respectively.

## 3 RESULTS

### 3.1 The expression of m5C regulators was different between BRCA and normal breast tissues

**FIGURE 1:**
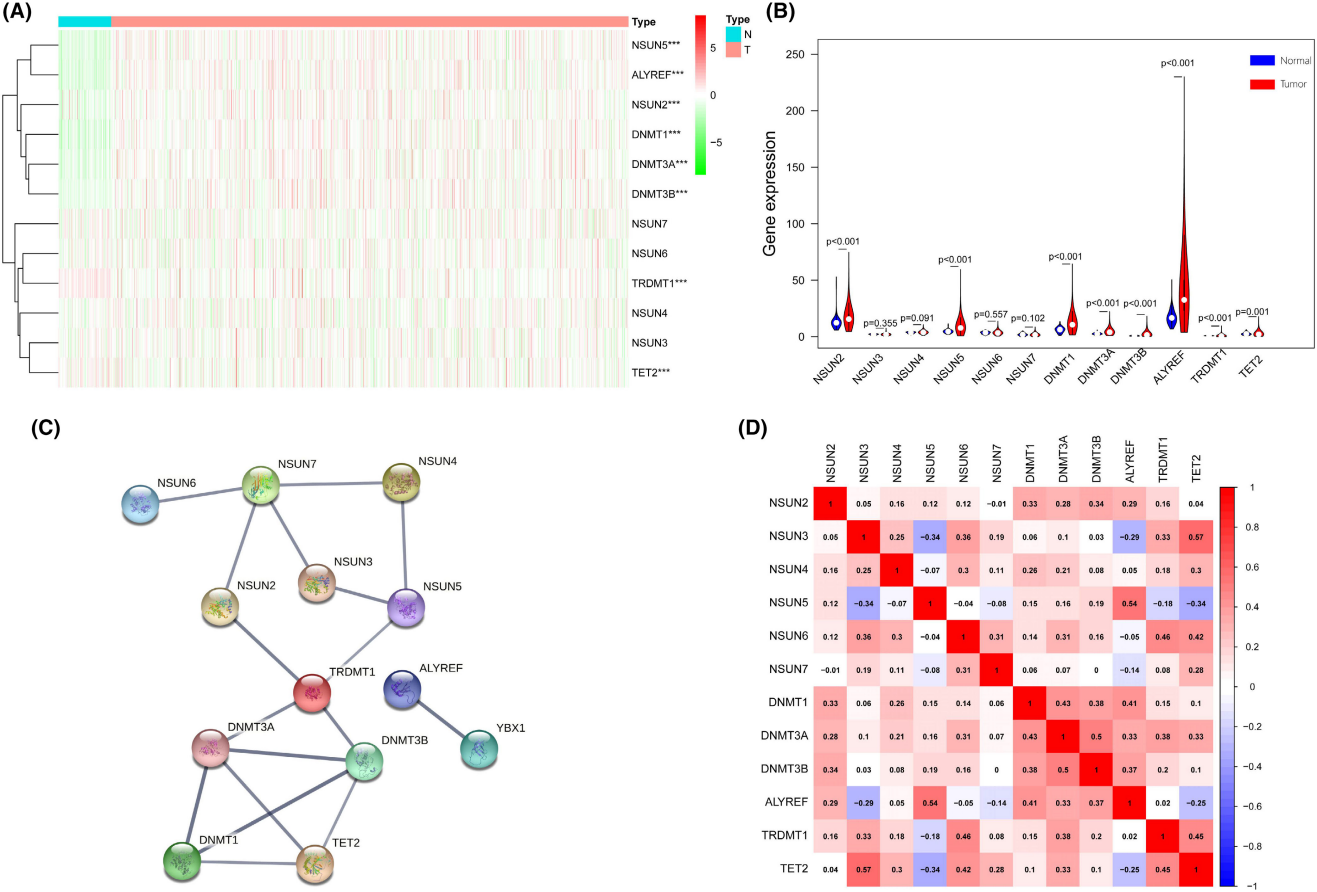
Differentially expressed m5C regulators between breast cancer tissues and nontumor tissues: (A) Heatmap of m5C regulators. The depth of green indicates the level of low expression, and the depth of red indicates the level of high expression; *** indicates *p* < 0.001. (B) Vioplot for the m5C regulators. The blue column indicates normal tissue, and the red column indicates tumor tissue. (C) Protein–protein interaction (PPI) network analysis of m5C regulators in BRCA. (D) Coexpression analyses for m5C regulators. The red color indicates a positive correlation, and the blue color indicates a negative correlation.

The RNA‐seq and clinical data, including 113 normal adjacent tissues and 1109 BRCA tumor tissues, were derived from the TCGA database. A heatmap of m5C regulators and a vioplot for their expression were generated and are presented in Figure 1A,B. The results showed that the expression levels of *NSUN2*, *NSUN5*, *DNMT1*, *DNMT3A*, and *DNMT3B* were significantly upregulated (*p* < 0.05), while the expression levels of *TRDMT1* and *TET2* were significantly downregulated (*p* < 0.05). Next, the protein–protein interaction network was generated based on the STRING database (Figure 1C) and suggested that *DNMT3A*, *DNMT3B*, *NSUN7*, and *TRDMT1* were the top four most strongly connected PPI nodes; they each had four edges with other genes. This correlation was validated by coexpression analysis, which was further quantified by Pearson's correlation coefficient (Figure 1D). The results indicated that expression levels of TET2 NSUN3, ALYREF, NSUN5, DNMT3A, and DNMT3B were strongly correlated (correlation coefficient ≥0.5), while those of NSUN2, NSUN4, NSUN6, NSUN7, DNMT1, and TRDMT1 were correlated with a lower coefficient (0.5 > correlation coefficient ≥0.3). The above results indicated that the breast m5C regulator genes had significant internal correlations.

### 3.2 Identification of m5c‐related lncRNAs and development of the prognostic signature model

**FIGURE 2:**
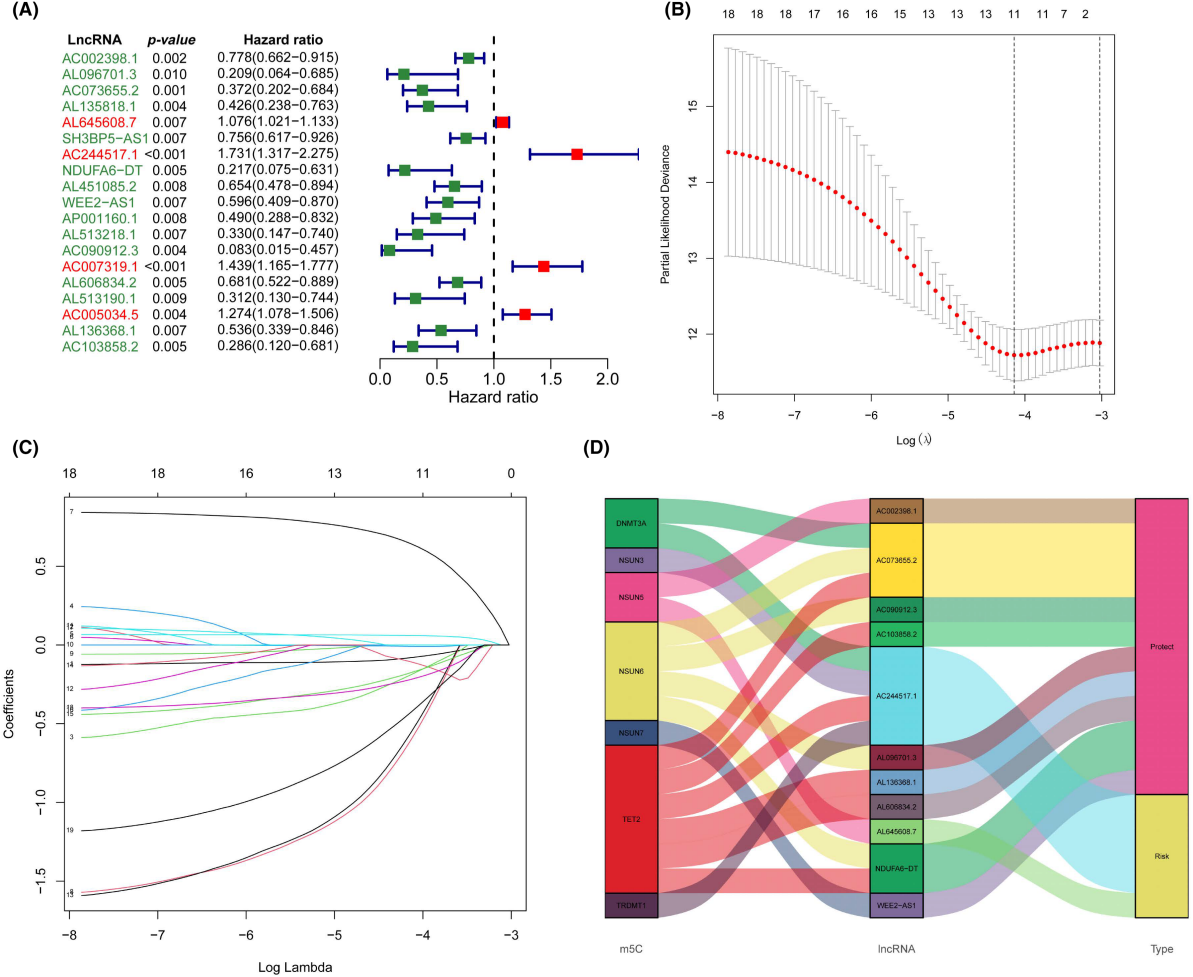
Key prognostic‐related lncRNAs and construction of the prognostic risk signature: (A) Forest plot of 19 prognostic‐related lncRNAs. Red‐colored lncRNAs indicate a risk‐related factor, and green‐colored lncRNAs indicate a protection‐related factor. (B) Lasso Cox regression of 11 lncRNAs used in the prognostic risk model. (C) Lasso filter variables. (D) Sankey plot demonstrating the relationship between the m5C regulators and m5C‐related prognostic signature lncRNAs.

LncRNAs that significantly correlated with one or more of the m5C regulators (|correlation coefficient| >0.3 and *p* value <0.001) were identified as m5C‐related lncRNAs. As suggested in Figure 2A, we performed univariate Cox regression analysis and identified 19 m5c‐related lncRNAs that were strongly associated with prognosis (*p* value <0.01). Lasso Cox regression analysis was implemented to construct the prognostic signature model including 11 prognostically relevant m5C‐related lncRNAs and achieved the following equation = −0.0807* *AC002398.1–0*.1063* *AL096701.3–0*.1904* *AC073655.2*+ 0.0602* *AL645608.7*+ 0.6444* *AC244517.1–0*.6082* *NDUFA6‐DT*‐0.0088* *WEE2‐AS1‐0*.561* *AC090912.3–0*.1767* *AL606834.2–0*.2194* *AL136368.1–0*.4299* *AC103858.2* (Figure 2B,C). Using this equation, the median risk score in the training group was 0.464675, which was set as the cutoff point of the high‐ and low‐risk groups in the training group and testing group of BRCA patients. The Sankey plot demonstrated the relationship among the m5C regulators, prognosis‐relevant m5C‐related lncRNAs and the impact of these lncRNAs on disease prognosis (Figure 2D). As suggested in Figure 2E–J, more death events occurred among patients with increased risk scores. In addition, lncRNAs that were associated with poor prognosis were highly expressed in the high‐risk group, while protective lncRNAs were highly expressed in the low‐risk group.

### 3.3 The prognostic value of the m5C‐related lncRNA prognostic signature model

**FIGURE 3:**
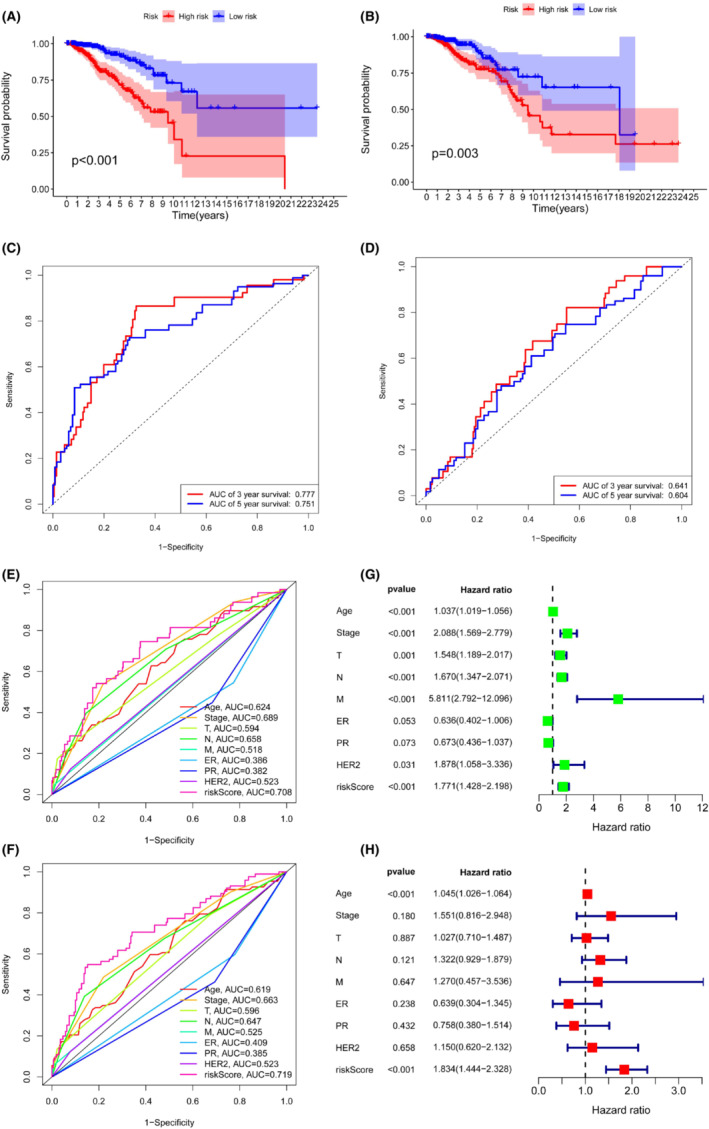
Performance and validation of the prognostic risk model: (A) Kaplan–Meier analysis of the training group. (B) Kaplan–Meier analysis of the testing group. (C) The ROC curve of overall survival for the training group. (D) The ROC curve of overall survival for the testing group. (E) Multivariate ROC analysis at 3 years. (F) Multivariate ROC analysis at 5 years. (G, H) Univariate and multivariate Cox regression analysis of clinical characteristics and risk scores.

**FIGURE 4:**
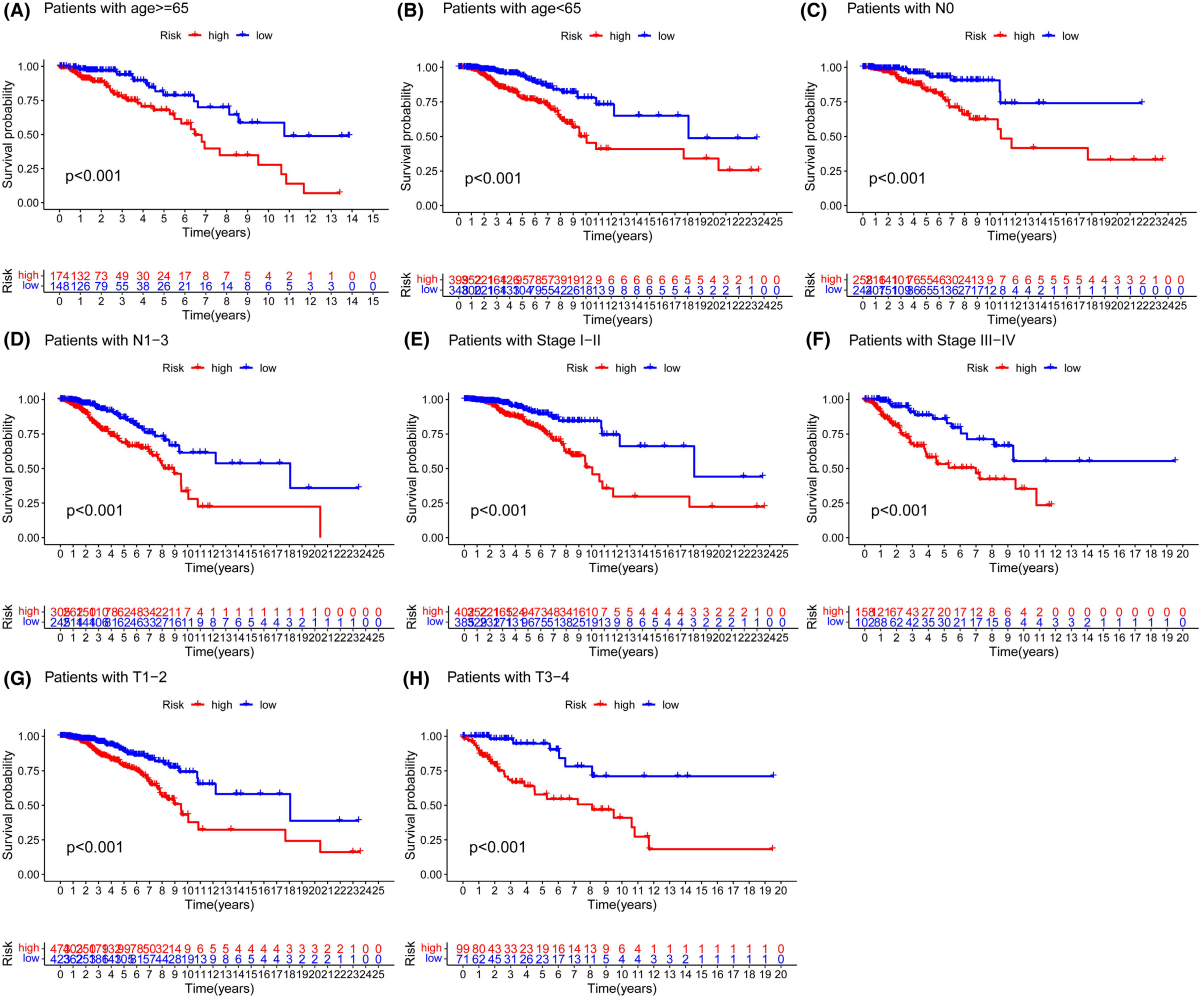
Clinical subgroup analysis of the prognostic model: (A, B) age, (C, D) stage, (E, F) T stage, and (G, H) N stage.

Kaplan–Meier survival analysis curves indicated that patients with high‐risk scores had significantly worse overall survival than patients with lower risk scores in the training group (Figure 3A, *p* < 0.001) and the testing group (Figure 3B, *p* = 0.003). The time‐dependent ROC analysis demonstrated that the 3‐year and 5‐year area under the curve (AUC) values for this novel model were 0.777 and 0.751, respectively, in the training group and 0.641 and 0.604, respectively, in the testing group (Figure 3C,D). Multivariate ROC analysis including several clinical characteristics and risk scores showed that the AUCs of 3‐year and 5‐year survival were 0.708 and 0.719, respectively, which were higher than those of other clinical characteristics (Figure 3E,F). These results indicated that the prognostic signature model had the highest prognostic value compared to other clinical characteristics. Univariate Cox regression and multivariate Cox regression analyses were then conducted to probe whether the prognostic signature model was an independent risk factor associated with the development of BRCA. The results showed that increased risk score (95% CI HR: 1.444–2.328; HR = 1.834; *p* < 0.001) and age (95% CI HR: 1.026–1.064; HR = 1.045; *p* < 0.001) were both independent risk factors associated with the development of BRCA (Figure 3G,H). Furthermore, the proposed prognostic model was further verified in different subgroups (Figure 4).

### 3.4 Construction and validation of the nomogram

**FIGURE 5:**
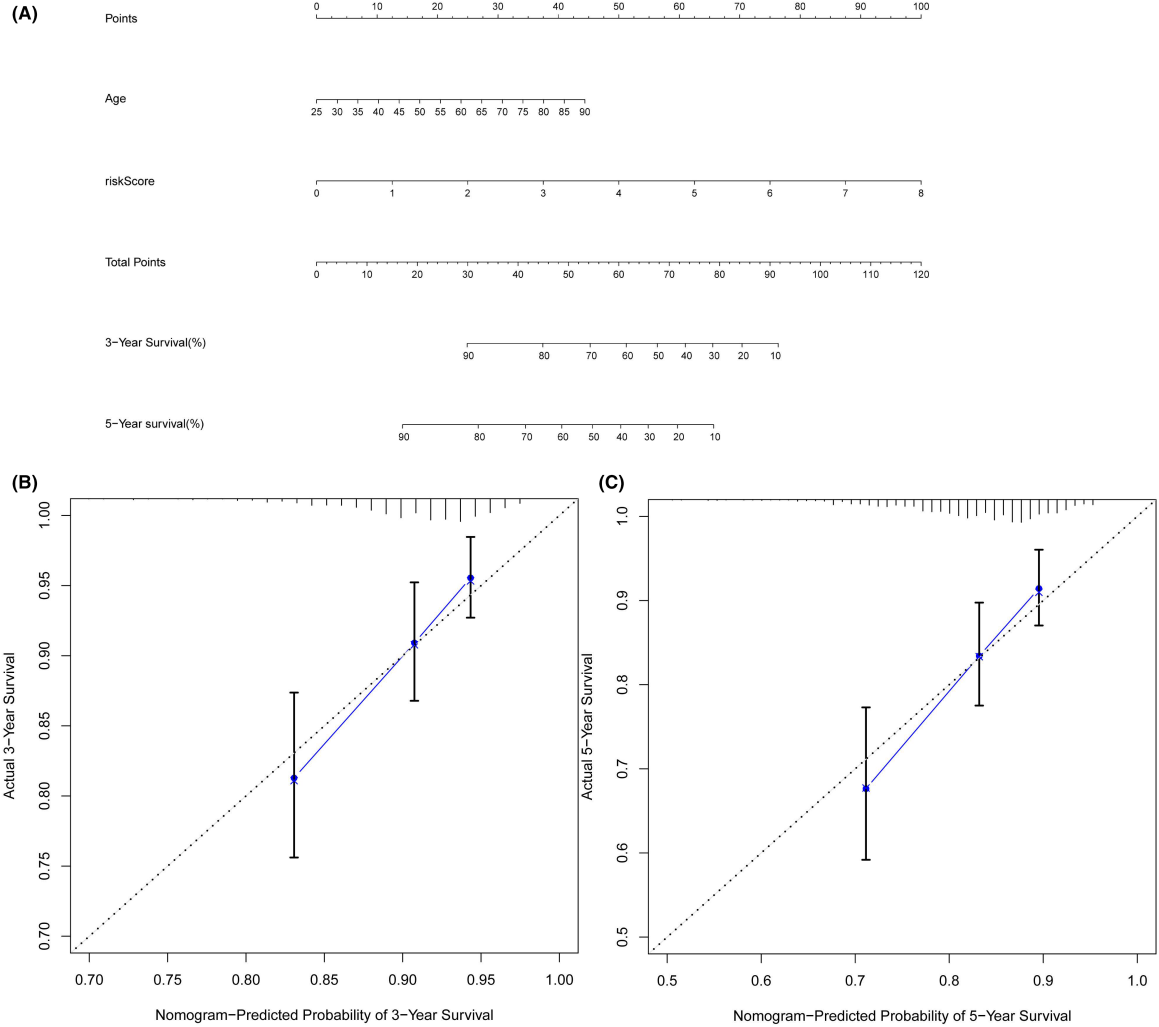
Nomogram and calibration curve of prognostic risk model: (A) Nomogram to predict 3‐ and 5‐year overall survival of breast cancer (BRCA) patients. (B, C) Calibration curves for 3‐year (B) and 5‐year (C) survival.

The nomogram, including independent prognostic‐related factors (model risk score and age) and calibration curves presenting the relationship between the actual prediction result (dotted line) and the ideal prediction result (blue dot with black solid line), is presented in Figure 5A–C. The c‐index of the nomogram was 0.707 with a standard error (SE) of 0.027.

### 3.5 Evaluation of the association between the m5C‐related prognostic signature model and immune cell infiltration

**FIGURE 6:**
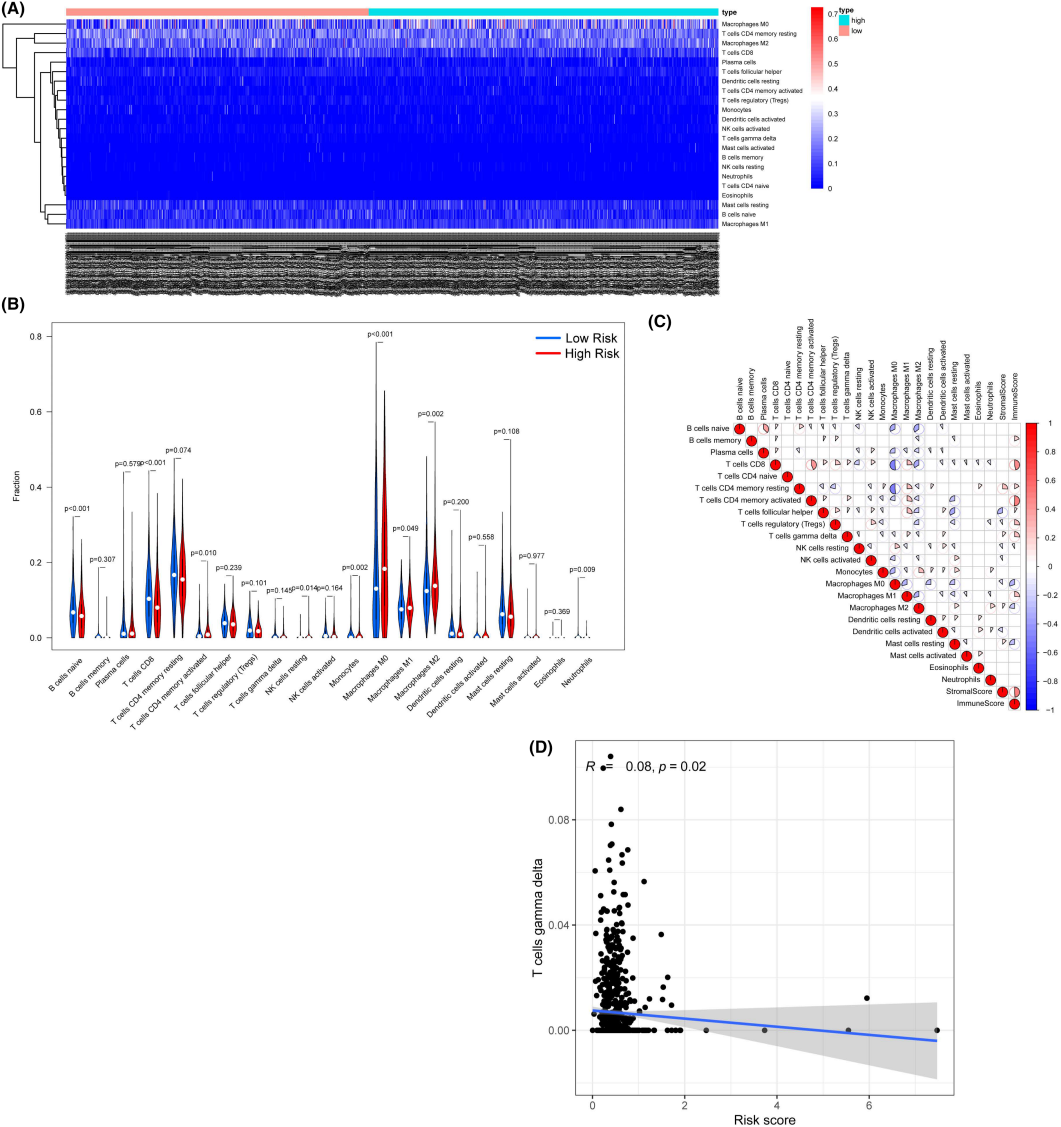
Immune cell infiltration analysis: (A) Heatmap of immune cell abundances. (B) Vioplot for the immune cell abundances in the high‐ and low‐risk groups.

Based on the *CIBERSORT* algorithm, we measured the infiltration of 21 types of TIICs (CD4 naive T cells excluded) and compared them between patients with high‐risk and low‐risk scores (Figure 6A,B). In the high‐risk group, the infiltration levels of activated memory CD4 T cells, resting NK cells, and M0‐M2 macrophages were significantly increased (*p* < 0.05), while the infiltration levels of naive B cells, CD8 T cells, monocytes, and neutrophils were significantly decreased (*p* < 0.05). We also assessed the correlations between immune cell infiltration and immune/stromal/ESTIMATE scores (Figure 6C). Our data suggested that the infiltration level of M0 macrophages was strongly negatively correlated with the infiltration levels of resting CD4 memory T cells and activated CD4 memory T cells (correlation coefficient ≥0.5), while the infiltration level of activated CD4 memory T cells was strongly positively correlated with immune scores (correlation coefficient ≥0.5). Next, Spearman's test was performed, and the results showed that the infiltration levels of a total of 10 types of TIICs were associated with risk scores (Figure 6D).

### 3.6 Association between the m5C‐related lncRNA signature and sensitivity to chemotherapy

**FIGURE 7:**
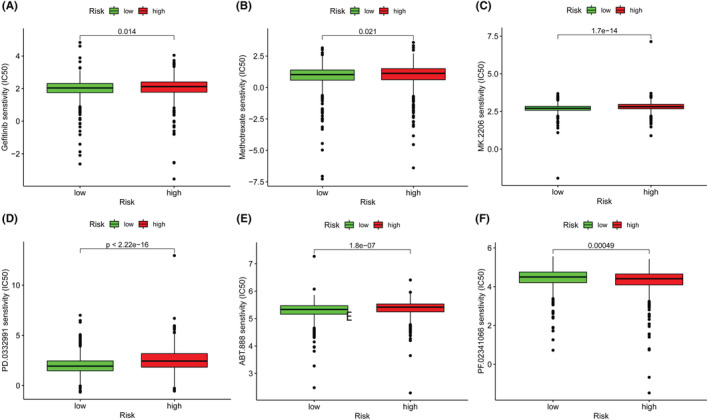
Chemotherapy drug IC50 levels for the lncRNA signature: (A) gefitinib, (B) methotrexate, (C) MK.2206, (D) palbociclib, (E) veliparib, and (F) crizotinib.

The IC50 levels of 137 different chemotherapy drugs were extracted from the dataset, and 77 of them were significantly different between the high‐risk group and the low‐risk group (*p* < 0.05; Table S3). For instance, the IC50 levels of gefitinib, methotrexate, MK 2206, palbociclib, and veliparib were higher, while that of crizotinib was significantly lower in the high‐risk group than in the low‐risk group (Figure 7).

### 3.7 Pathways associated with the m5C‐related lncRNA signature

**FIGURE 8:**
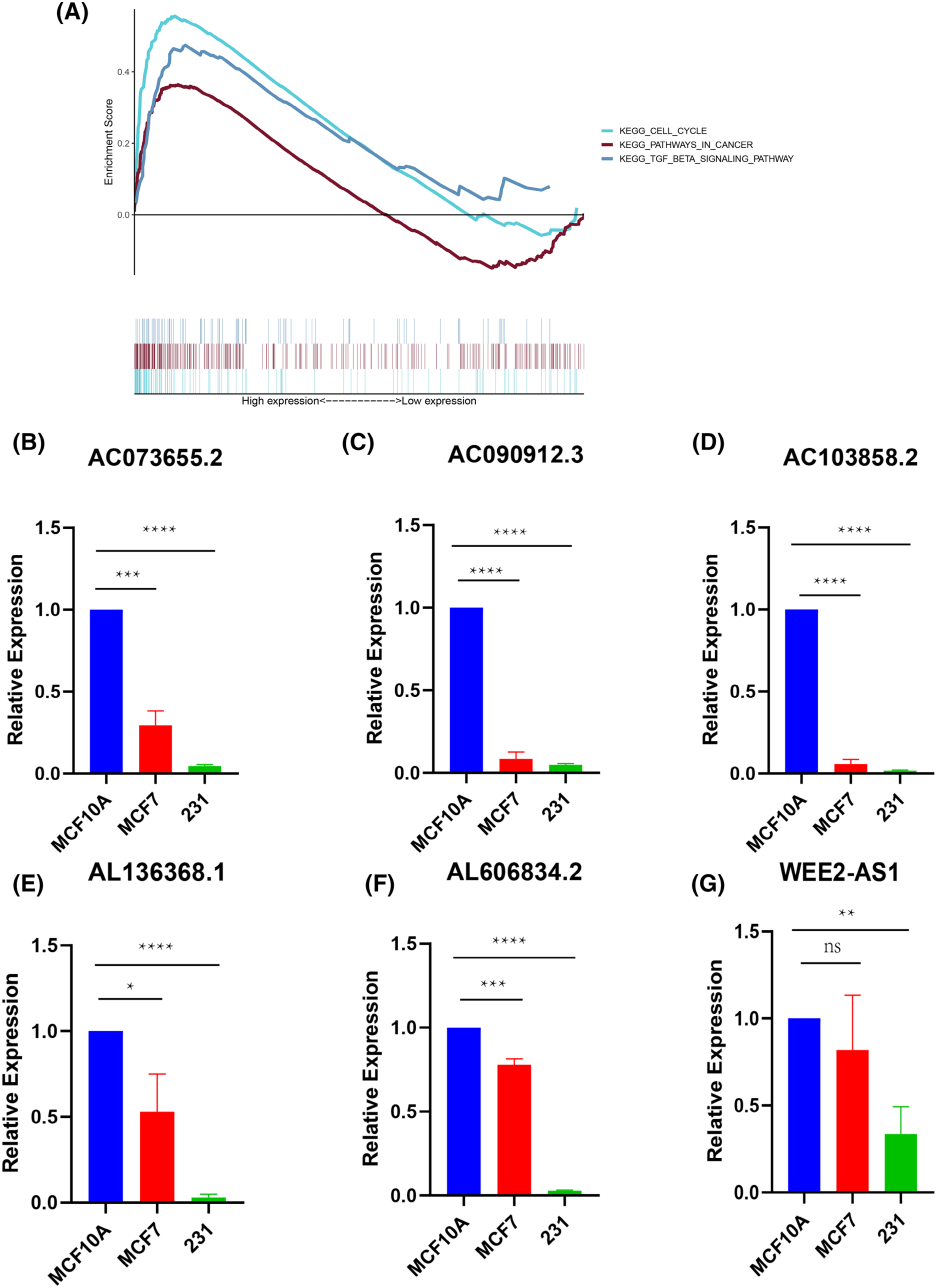
(A) Gene set enrichment analysis; (B–G) qRT–PCR analysis for the lncRNA signature. (ns: not significant, *: *p* < 0.05, **: *p* < 0.01, ***: *p* < 0.001, ****: *p* < 0.0001).

In the high‐risk group, the genes were found to be associated with cell cycle and cancer pathways, including KEGG, KEGG‐TGF beta, and KEGG PATHWAY IN CANCER (Figure 8A).

### 3.8 Expression level of m5C‐related lncRNAs in breast cancer cells

We performed qRT–PCR to determine the lncRNA expression levels. The results showed that most signature lncRNAs were expressed at a relatively low level in breast cancer cells. The expression levels of some lncRNAs (AL645608.7, NDUFA6‐DT, AL096701.3, AC244517.1, and AC002398.1) could not be determined due to a lack of primer sequences (Figure 8B–G).

### 3.9 High expression of the lncRNA WEE2‐AS1 was associated with better prognosis in the GEO dataset

**FIGURE 9:**
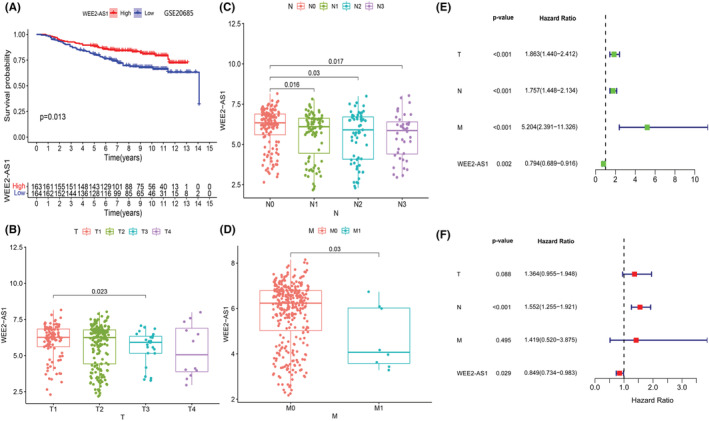
Validation of the prognostic value and independence of the lncRNA WEE2‐AS1 in the external dataset GSE 20685: (A) Kaplan–Meier survival analysis of the lncRNA WEE2‐AS1 in breast cancer patients. (B) Boxplot for WEE2‐AS1 among patients with different T stages in dataset GSE20685. (C) Boxplot for WEE2‐AS1 among patients with different N stages in dataset GSE20685. (D) Boxplot for WEE2‐AS1 among patients with different M stages in dataset GSE20685. (E, F) Univariate and multivariate Cox regression analyses of clinical characteristics and WEE2‐AS1 expression.

The median expression of lncRNA WEE2‐AS1 was set as the cutoff point for the high‐ and low‐expression groups. As the Kaplan–Meier survival curve shows in Figure 9A, the patients with low expression of WEE2‐AS1 had a worse prognosis (*p* = 0.013). This result validated the conclusion of Cox regression analysis in the TCGA dataset. We also explored the relationship between clinical characteristics and the expression of WEE2‐AS1 in the GSE20685 dataset. The expression of WEE2‐AS1 was significantly correlated with T stage, N stage and M stage (Figure 9B–D, *p* < 0.05). The results of univariate Cox regression analysis and multivariate Cox regression analysis indicated that the expression of the lncRNA WEE2‐AS1 and N stage was independently associated with the prognosis of breast cancer (Figure 9E,F, *p* < 0.05). The C‐index comparison figure shown in Figure S1 shows the superiority of our lncRNA signature to others. Our m5C‐lncRNA signature's C‐index was 0.686, which was better than Huang's m5C‐lncRNA signature (C‐index = 0.602),^19^ Jin's immune‐lncRNA signature (C‐index = 0.675),^20^ and Zhang's m6A‐lncRNA signature (C‐index = 0.65).^21^

## 4 DISCUSSION

The overall survival of BRCA has been significantly improved due to the innovation of targeted therapy and radiotherapy. Nevertheless, some critical problems, such as drug resistance in hormone receptor‐positive breast cancer and the clinical challenge of triple‐negative breast cancer, remain to be solved. Thus, discoveries of novel biomarkers and therapeutic approaches are urgently needed. LncRNAs are a group of noncoding RNAs that are more than 200 nucleotides in length and regulate gene expression at the transcriptional and posttranscriptional levels.^20^ It is known that abnormal lncRNA expression participates in the progression of tumors in several types of cancer, especially breast cancer and lung cancer.^21^, ^22^ Previous studies have suggested the potential prognostic value of m5C modification‐related regulators in various cancers.^23^, ^24^, ^25^

In our study, 13,162 m5C‐related lncRNAs were identified from the TCGA‐BRCA dataset, 11 of which we incorporated into the prognostic signature model. Our results showed that the risk calculated by the expression of the above lncRNAs was associated with prognosis. Furthermore, this prognostic signature was visualized by nomogram, which is useful in daily clinical work.

The expression of some lncRNAs has been identified in other types of tumors. For instance, the expression of NDUFA6‐DT was reported to be a protective factor in glioblastoma.^26^ Low expression of WEE2 antisense RNA 1 (WEE‐AS1) suppresses the migration and invasion of triple‐negative breast cancer cells through the WEE‐AS1‐miR32‐5p/TOB1 axis.^27^ In addition, the oncogenic role of WEE‐AS1 was also identified in glioblastoma, which acts as a sponge for miR‐520f‐3p.^28^ However, the remaining lncRNAs are rarely reported.

The drug sensitivity results obtained in this study might provide additional clinical relevance for our model. Our data demonstrated that the sensitivities to some chemotherapy drugs, such as methotrexate and palbociclib, were different between patients with different risk scores. Some previous studies indicated that the differential expression of lncRNAs could affect the growth of breast tumors under treatment with novel drugs, such as palbociclib and gefitinib.^29^ However, these tests are not routinely performed clinically.

We also examined immune cell infiltration between patients with different risk scores. In particular, the infiltration of M2 macrophages was more abundant in the high‐risk group. Tumor‐associated macrophages (TAMs) are important tumor‐promoting cells in the tumor microenvironment (TME) and contribute to breast tumor growth, invasion, and metastasis.^30^, ^31^ Some previous studies reported that lncRNAs and miRNAs regulate tumor progression by promoting M2 macrophage phenotype polarization, preventing macrophage recruitment, and disrupting communication between macrophages.^21^, ^32^ The associations between TAMs and m5C‐related lncRNAs should be further studied in the future.

Finally, GSEA proposed several pathways that might be involved in the associations between lncRNAs and disease prognosis, including the TGF‐β signaling pathway, which is known to be associated with the 5‐methylcytosine “eraser” TET2. The deletion of TET2 leads to Foxp3 hypermethylation and Treg cell damage, while increased expression of TET2 can maintain the methylation balance through the TGF‐β signaling pathway and IL‐2 signaling pathway.^33^ Knockdown of the senescent breast cancer cell 5‐methylcytosine writers “DNMT2” and “TRDMT1” resulted in a prolonged G2/M phase in the cell cycle.^33^

There are several limitations of this study. First, the external validation of the m5C‐related lncRNA signature is missing due to the different microarray chip platforms and lack of complete lncRNA expression data in the Gene Expression Omnibus (GEO) database. Furthermore, the m5C level of m5C‐related lncRNAs should be determined by several experiments, such as m5C‐MeRIP‐seq and m5C‐RNA‐BisSeq. More in vitro validation with samples obtained from BRCA patients and animal models should be performed in the future.

## 5 CONCLUSION

In conclusion, we constructed an m5C‐related lncRNA‐based risk score signature and a nomogram to predict the prognosis of BRCA. Furthermore, this study revealed associations between m5C‐related lncRNAs and immune cell infiltration as well as chemotherapy drug sensitivity.

## AUTHOR CONTRIBUTIONS

**Gaoran Xu:** Conceptualization (lead); writing – review and editing (lead). **Chengxin Li:** Conceptualization (equal); data curation (lead); writing – original draft (lead); writing – review and editing (equal). **Ziyang Di:** Conceptualization (equal); project administration (lead). **Yalong Yang:** Data curation (equal). **Leilei Liang:** Formal analysis (equal). **Qianqian Yuan:** Data curation (equal); formal analysis (equal); validation (equal). **Qian Yang:** Methodology (equal); writing – original draft (equal); writing – review and editing (equal). **Xingxing Dong:** Supervision (equal); validation (equal). **Siguang Xu:** Project administration (equal); writing – original draft (equal); writing – review and editing (equal). **Gaosong Wu:** Conceptualization (lead); writing – review and editing (lead).

## FUNDING INFORMATION

This work was supported by the major subject fund of Zhongnan Hospital of Wuhan University (XKJS202015).

## CONFLICT OF INTEREST

The authors declare that they have no conflict of interest.

## CONSENT FOR PUBLICATION

All authors have consented for the publication.

## ETHICS STATEMENT

This study was based on a TCGA dataset and its primary study had received ethical approval.

## Supporting information


Figure S1

Tables S1‐S3

## Data Availability

The data that support the findings of this study are available in The Cancer Genome Atlas databases at https://www.cancer.gov/about‐nci/organization/ccg/research/structural‐genomics/tcga, reference number: TCGA‐BRCA.
