# Supplementary figures and images for: Development of the expression and prognostic significance of m5C‐related LncRNAs in breast cancer
Source: Cancer Med. 2022 Dec 4;12(6):7667–81. doi: 10.1002/cam4.5500 (PMC10067052; doi:10.1002/cam4.5500)

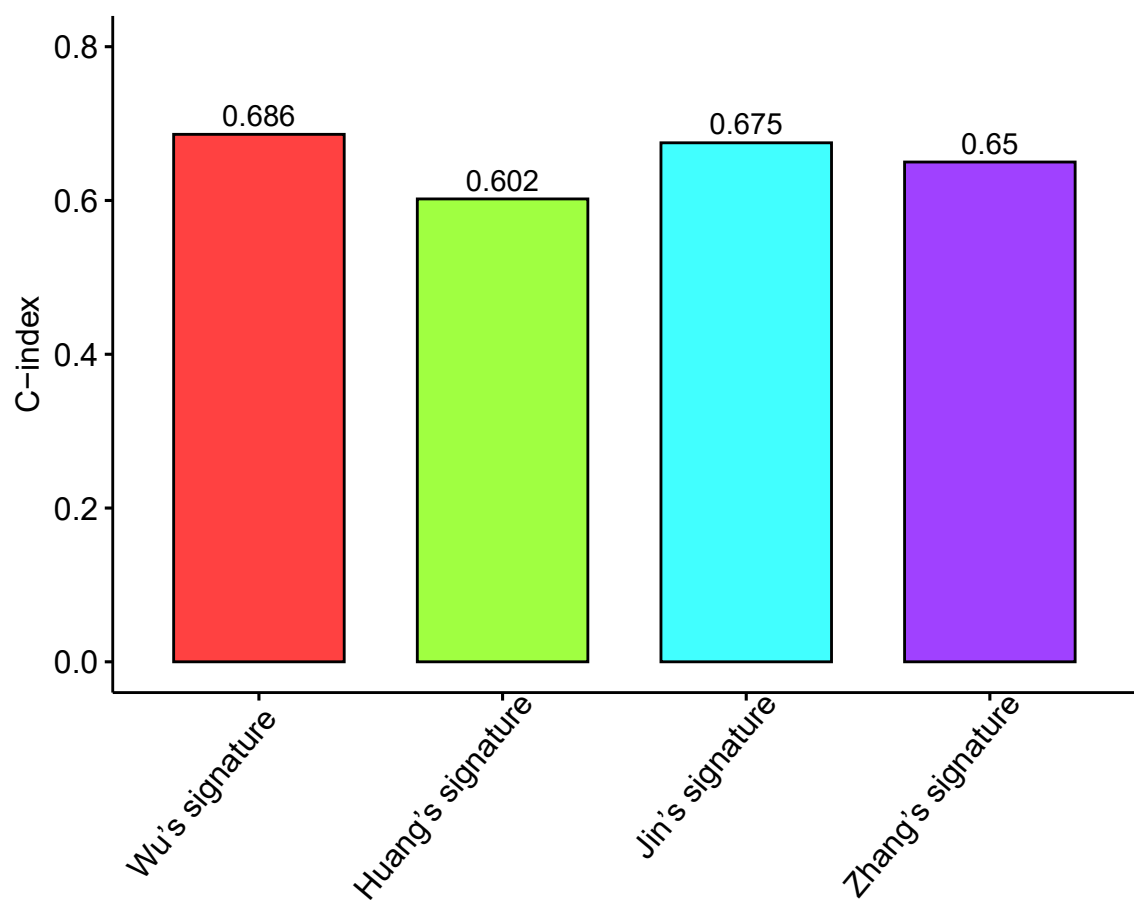

Supplement: Supplementary file 1 — Figure S1 [file CAM4-12-7667-s002.pdf]
