# Supplementary material for: Development of the expression and prognostic significance of m5C‐related LncRNAs in breast cancer
Source: Cancer Med. 2022 Dec 4;12(6):7667–81. doi: 10.1002/cam4.5500 (PMC10067052; doi:10.1002/cam4.5500)
Supplement: Supplementary file 2 — Tables S1‐S3 [file CAM4-12-7667-s001.docx]

**Supplementary Table 1** Clinical characteristic details of training group and testing group patients

| Covariates | Type | Total | Test | Train |
| --- | --- | --- | --- | --- |
| Age | <=65 | 768(71.84%) | 380(71.29%) | 388(72.39%) |
| Age | >65 | 301(28.16%) | 153(28.71%) | 148(27.61%) |
| Stage | Stage I-II | 787(73.62%) | 407(76.36%) | 380(70.9%) |
| Stage | Stage III-IV | 260(24.32%) | 112(21.01%) | 148(27.61%) |
| Stage | unknow | 22(2.06%) | 14(2.63%) | 8(1.49%) |
| T | T1-2 | 896(83.82%) | 448(84.05%) | 448(83.58%) |
| T | T3-4 | 170(15.9%) | 83(15.57%) | 87(16.23%) |
| T | unknow | 3(0.28%) | 2(0.38%) | 1(0.19%) |
| M | M0 | 890(83.26%) | 457(85.74%) | 433(80.78%) |
| M | M1 | 22(2.06%) | 8(1.5%) | 14(2.61%) |
| M | unknow | 157(14.69%) | 68(12.76%) | 89(16.6%) |
| N | N0 | 502(46.96%) | 262(49.16%) | 240(44.78%) |
| N | N1-3 | 550(51.45%) | 266(49.91%) | 284(52.99%) |
| N | unknow | 17(1.59%) | 5(0.94%) | 12(2.24%) |

**Supplementary Table 2** The primer sequences involved in this study

| Primer | 5' to 3' |
| --- | --- |
| AC073665.2-F | GAGGCATCAGTGTGCGAAGGTC |
| AC073665.2-R | AAGCACGGTCCTTCAAAGATGGC |
| AC090912.3-F | AGAGAATGAGGAGGGTCGGATCT |
| AC090912.3-R | TCCTCTGCATTGAAGAGATGAAGTCT |
| AC103858.2-F | CCGTTGCCCACTTTCTTCCCTTC |
| AL103858.2-R | GTCCCTTCCCTGAGCCTCCTTAG |
| AL136368.1-F | AGGAGGAATCGGACAGGCTGATC |
| AL136368.1-R | TCACAGGGCGTTCTGCAAACTC |
| AL606834.2-F | AGGACAAAGGAGAGGGAAGAGTCAG |
| AL606834.2-R | ACCGCTGGGTTCATTCACTCATTC |
| WEE2-AS1-F | CCTCTCCCGACCTGTGATAC |
| WEE2-AS1-R | GCTTGCTGCTTTGCTGCTTCTATG |
| GAPDH-F | GGTGTGAACCATGAGAAGTATGA |
| GAPDH-R | GAGTCCTTCCACGATACCAAAG |

**Supplementary Table 3** The result of drug sensitivity analysis

| Drug | *p*-value | Risk group with higher IC50 |
| --- | --- | --- |
| A.443654 | 2.4E-02 | low |
| A.770041 | 1.3E-03 | low |
| ABT.263 | 7.7E-03 | low |
| AG.014699 | 4.1E-03 | high |
| AMG.706 | 1.6E-08 | high |
| AS601245 | 2.3E-04 | high |
| ATRA | 6.8E-12 | high |
| AZD 0530 | 2.4E-07 | low |
| AZD 6244 | 2.6E-02 | low |
| AZD 7762 | 3.6E-06 | low |
| AZD 8055 | 1.1E-04 | high |
| Bexarotene | 2.1E-02 | low |
| BI.D1870 | 9.5E-03 | low |
| Bicalutamide | 2.2E-05 | low |
| BIRB.0796 | 1.4E-09 | high |
| BMS.754807 | 2.8E-02 | low |
| Bosutinib | 1.2E-02 | high |
| BX.795 | 1.2E-06 | low |
| Camptothecin | 1.9E-03 | high |
| CCT007093 | 4.6E-08 | high |
| CCT018159 | 7.8E-05 | high |
| CEP 701 | 4.2E-02 | high |
| CGP 082996 | 1.8E-03 | low |
| CMK | 8.7E-15 | low |
| Cytarabine | 2.0E-04 | high |
| Dasatinib | 4.6E-03 | low |
| DMOG | 3.0E-05 | low |
| EHT 1864 | 3.6E-12 | high |
| GDC 0449 | 9.8E-04 | high |
| Gefitinib | 1.4E-02 | high |
| GNF 2 | 3.1E-05 | low |
| GSK 650394 | 4.6E-04 | high |
| GW 441756 | 3.9E-02 | high |
| IPA 3 | 1.3E-12 | high |
| JNJ 26854165 | 4.3E-06 | low |
| KIN001 135 | 4.4E-06 | low |
| Lenalidomide | 1.2E-05 | high |
| Metformin | 1.0E-14 | high |
| Methotrexate | 2.1E-02 | high |
| MK 2206 | 1.7E-14 | high |
| Nilotinib | 8.9E-04 | high |
| NSC 87877 | 4.5E-04 | low |
| NU 7441 | 5.0E-03 | low |
| Nutlin 3a | 1.6E-13 | high |
| NVP BEZ235 | 2.6E-03 | high |
| NVP TAE684 | 3.1E-05 | low |
| OSI 906 | 3.0E-03 | low |
| PAC 1 | 3.8E-02 | high |
| Palbociclib | <2.2E-16 | high |
| Parthenolide | 6.7E-05 | low |
| Pazopanib | 5.6E-05 | low |
| PD 173074 | 2.9E-02 | high |
| PF 02341066 | 4.9E-04 | low |
| PHA 665752 | 1.1E-06 | low |
| Pyrimethamine | 4.0E-02 | high |
| RDEA119 | 2.9E-02 | low |
| Roscovitine | 3.7E-06 | high |
| Salubrinal | 2.4E-05 | high |
| SB590885 | 4.4E-10 | high |
| Shikonin | 8.1E-06 | high |
| SL.0101.1 | 3.4E-02 | high |
| Sorafenib | 3.3E-02 | low |
| Temsirolimus | 1.9E-09 | high |
| Thapsigargin | 2.0E-08 | low |
| Tipifarnib | 3.2E-05 | high |
| Veliparib | 1.8E-07 | high |
| Vinblastine | 8.7E-07 | low |
| Vinorelbine | 1.4E-02 | high |
| Vorinostat | 3.5E-05 | high |
| VX.702 | 1.3E-02 | high |
| WH.4.023 | 1.6E-04 | low |
| WZ.1.84 | 3.3E-07 | low |
| X17.AAG | 2.3E-02 | low |
| XMD8.85 | 5.1E-03 | low |
| Z.LLNle.CHO | 5.8E-10 | low |
| ZM.447439 | 1.6E-02 | low |
